# ﻿A new species of the *Pholcusphungiformes* species group (Araneae, Pholcidae) from Liaoning, China, with identification keys to four closely related species

**DOI:** 10.3897/zookeys.1193.115640

**Published:** 2024-03-06

**Authors:** Ludan Zhang, Bing Wang, Qiaoqiao He, Zhiyuan Yao

**Affiliations:** 1 College of Life Science, Shenyang Normal University, Shenyang 110034, Liaoning, China Shenyang Normal University Shenyang China; 2 Liaoning Key Laboratory of Evolution and Biodiversity, Shenyang 110034, Liaoning, China Liaoning Key Laboratory of Evolution and Biodiversity Shenyang China; 3 Liaoning Key Laboratory for Biological Evolution and Agricultural Ecology, Shenyang 110034, Liaoning, China Liaoning Key Laboratory for Biological Evolution and Agricultural Ecology Shenyang China

**Keywords:** Biodiversity, daddy-long-legs spider, morphology, Northeast Asia, Pholcinae, taxonomy

## Abstract

A new species of pholcid spiders, *Pholcusfengmeii* Zhang, He & Yao, **sp. nov.** (♂♀), is described from Liaoning Province, China. The new species belongs to the speciose *phungiformes* species group. Taxonomic keys to four closely related species are provided.

## ﻿Introduction

**Figure 1.**
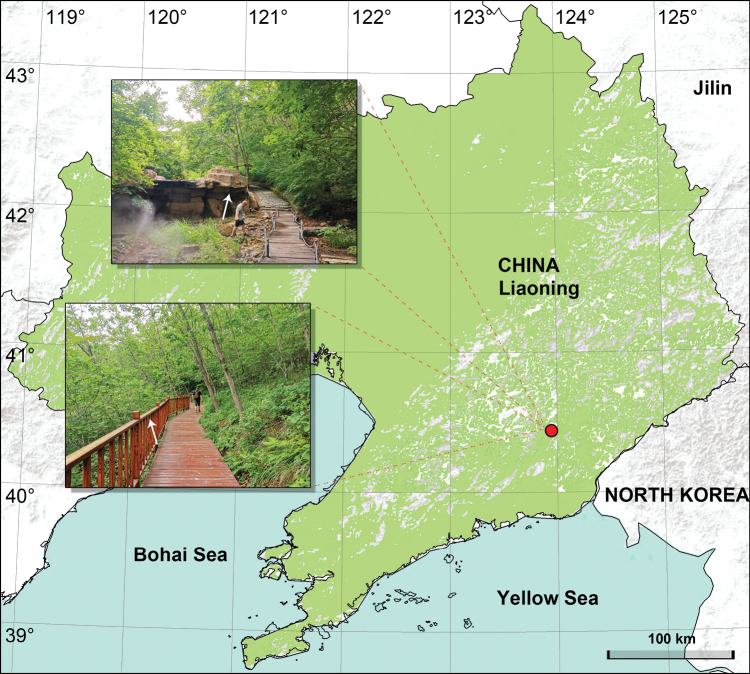
Distribution of *Pholcusfengmeii* sp. nov. from Liaoning, China. Arrows indicate habitats.

Pholcidae C.L. Koch, 1850 is one of the most species-rich spider families, with 1,946 extant species in 97 genera (WSC 2024). *Pholcus* Walckenaer, 1805 is the most diverse genus in the family, with 384 described species mainly distributed in the Afrotropical, Palaearctic, Indo-Malayan, and Australasian regions (Huber 2011; Yao and Li 2012; WSC 2024). The genus was split to 21 species groups by Huber (2011) and Huber et al. (2018), of which the *phungiformes* group is the most speciose and contains 108 species (Huber 2011; Wang et al. 2020; Yao et al. 2021; Lu et al. 2022; Zhao et al. 2023a, 2023b). Almost all species of this group are recorded from four mountain ranges: the Lüliang Mountains (9 spp.) and the Yanshan–Taihang Mountains (35 spp.) in North China, the Changbai Mountains (27 spp.) at the border between northeastern China and North Korea, and the Taebaek Mountains (44 spp.) on the Korean Peninsula (Jang et al. 2023). The only exception is *P.phungiformes* Oliger, 1983, which is known in the Maritime Territory, Sakhalin Island, and the Kurile Islands, Russia (Huber 2011). The Lüliang Mountains represent the westernmost limit of the distribution of the *phungiformes* group (Zhao et al. 2023b). The aim of this work is to describe a new species from Liaoning (Fig. 1), which occurs in the Changbai Mountain range in northeastern China. Taxonomic keys are provided to separate it from three other morphologically similar species, also occurring in Liaoning.

## ﻿Materials and methods

Specimens were examined and measured with a Leica M205 C stereomicroscope. The left male palp was photographed. The epigyne was photographed before dissection. The vulva was photographed after treating it in a 10% warm solution of potassium hydroxide (KOH) to dissolve soft tissues. Images were captured with a Canon EOS 750D wide zoom digital camera (24.2 megapixels) mounted on the stereomicroscope mentioned above and assembled using Helicon Focus v. 3.10.3 image-stacking software (Khmelik et al. 2005). All measurements are given in millimeters (mm). Leg measurements are shown as: total length (femur, patella, tibia, metatarsus, tarsus). Leg segments were measured on their dorsal side. The distribution map was generated with ArcGIS v. 10.2 (ESRI Inc.). The specimens studied are preserved in 75% ethanol and deposited in the College of Life Science, Shenyang Normal University (SYNU) in Liaoning, China.

Terminology and taxonomic descriptions follow Huber (2011) and Yao et al. (2015, 2021). The following abbreviations are used in the descriptions: **ALE** = anterior lateral eye, **AME** = anterior median eye, **PME** = posterior median eye, **L/d** = length / diameter ratio; used in the illustrations: **aa** = anterior arch, **b** = bulb, **da** = distal apophysis, **e** = embolus, **fa** = frontal apophysis, **pa** = proximo-lateral apophysis, **pp** = pore plate, **pr** = procursus, **u** = uncus.

## ﻿Taxonomic accounts

**Family Pholcidae C.L. Koch, 1850**

**Subfamily Pholcinae C.L. Koch, 1850**

### ﻿Genus Pholcus

Taxon classificationAnimaliaAraneaePholcidae

Walckenaer, 1805

B0359553-973C-54F6-92F4-60704E75D720

#### Type species.

*Araneaphalangioides* Fuesslin, 1775.

##### ﻿*Pholcusphungiformes* species group

The species group was recognized by Huber (2011). Currently, 23 species belonging to this group have been recorded from Liaoning Province. Of these, three species are similar to *P.fengmeii* sp. nov., and therefore we provide keys that allow distinguishing these four sibling species.

### ﻿Identification keys to four closely related species from Liaoning Province, China

Males

**Table:** 

1	Procursus with ventro-subdistal apophysis (e.g. arrow 2 in Fig. 4G); uncus not half-round (e.g. Fig. 4H)	**2**
–	Procursus without ventro-subdistal apophysis (Fig. 2C); uncus nearly half-round, with latero-median protrusion (arrow in Fig. 3C)	***P.fengmeii* sp. nov.**
2	Procursus with wide (length/width ratio: 2) prolatero-subdistal sclerite (arrow 1 in Fig. 4D); prolatero-subdistal sclerite with angular proximal apophysis (arrow 3 in Fig. 4D)	***P.phoenixus***
–	Procursus with narrow (length/width ratio: 4) prolatero-subdistal sclerite (e.g. arrow 1 in Fig. 4G); prolatero-subdistal sclerite without angular proximal apophysis (e.g. Fig. 4G)	**3**
3	Prolatero-subdistal sclerite of procursus curved (arrow 1 in Fig. 4A); procursus with short (as wide as long) and weakly sclerotized ventro-subdistal apophysis (arrow 2 in Fig. 4A); uncus medially strongly protruding and distally strongly curved (arrows 1, 2 in Fig. 4B)	***P.jiguanshan***
–	Prolatero-subdistal sclerite of procursus straight (arrow 1 in Fig. 4G); procursus with long (length/width ratio: 2) and strongly sclerotized ventro-subdistal apophysis (arrow 2 in Fig. 4G); uncus not protruding medially, distally slightly curved (arrows 1, 2 in Fig. 4H)	***P.yaoshan***

Females

**Table:** 

1	Anterior arch straight (Fig. 4F); pore plates nearly triangular (Fig. 4F)	***P.phoenixus***
–	Anterior arch curved (e.g. Fig. 3B); pore plates nearly elliptical or half-round (e.g. Fig. 4I, Fig. 3B)	**2**
2	Anterior arch laterally curved (Fig. 4I); pore plates nearly elliptical (Fig. 4I)	***P.yaoshan***
–	Anterior arch medially curved; pore plates nearly half-round or anteriorly wide and posteriorly narrow and pointed	3
3	Anterior arch medially strongly curved (bow-shaped; Fig. 3B); pore plates nearly half-round (Fig. 3B)	***P.fengmeii* sp. nov.**
–	Anterior arch medially slightly curved (ridge-shaped; Fig. 4C); pore plates nearly elliptical but anteriorly wide and posteriorly narrow and pointed (Fig. 4C)	***P.jiguanshan***

### ﻿ Pholcus fengmeii

Taxon classificationAnimaliaAraneaePholcidae

Zhang, He & Yao sp. nov.

3C79BD89-7110-536D-8D3C-E75E2DCAD00D

https://zoobank.org/6A3D5A94-C9FC-411E-8E5E-13E13B8AE018

Figs 2 , 3

#### Type material.

***Holotype***: ♂ (SYNU-Ar00357), **China**, **Liaoning**, Dandong, Fengcheng, Dalishu Village, Yaowanggu (40°26.30'N, 123°56.65'E, 298 m), 2 July 2023, Q. He & Z. Yao leg. ***Paratypes***: 2♂ (SYNU-Ar00358–59), 3♀ (SYNU-Ar00360–62), same data as for the holotype.

#### Etymology.

The specific name is dedicated to the late Deputy of China’s National People’s Congress, Fengmei Mao (1949–2014). Under the leadership of Fengmei Mao, the villagers of Dalishu in Liaoning Province were inspired to work hard and work smart, embarked on an entrepreneurial journey from 1980, and transformed their spartan hamlet into the prosperous and flourishing community it is today.

#### Diagnosis.

**Figure 2.**
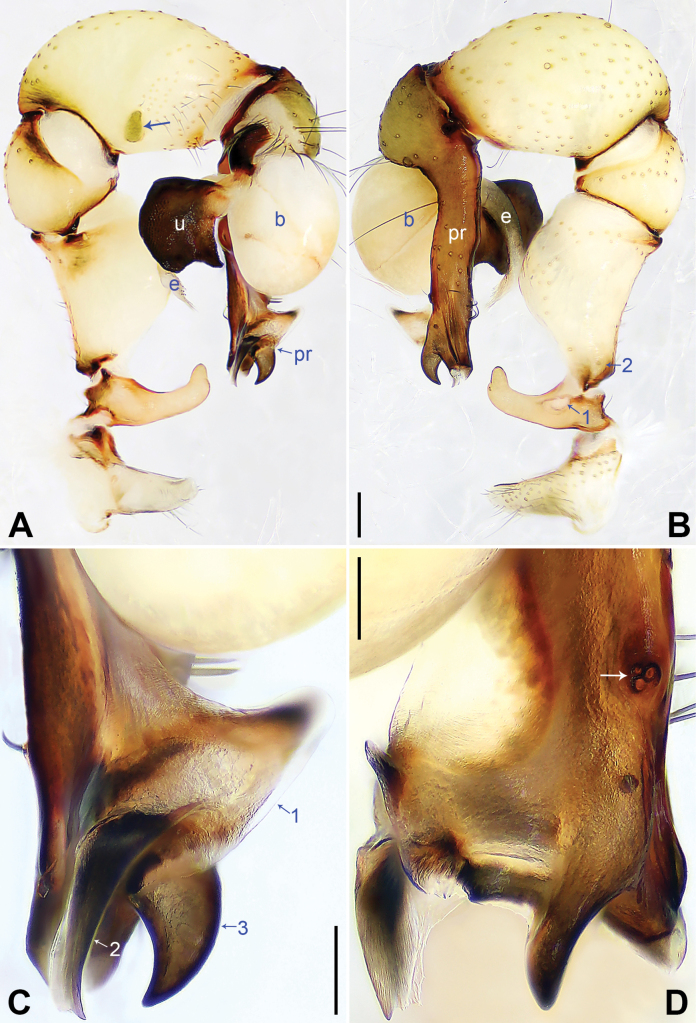
*Pholcusfengmeii* sp. nov., holotype male **A, B** palp (**A** prolateral view, arrow indicates prolatero-ventral protrusion **B** retrolateral view, arrow 1 indicates retrolaterally strongly bulged part, arrow 2 indicates retrolatero-proximal protrusion) **C, D** distal part of procursus (**C** prolateral view, arrow 1 indicates wide prolatero-subdistal sclerite, arrow 2 indicates curved proximal apophysis, arrow 3 indicates curved distal apophysis **D** dorsal view, arrow indicates dorsal spines). Abbreviations: b = bulb, e = embolus, pr = procursus, u = uncus. Scale bars: 0.20 mm (**A, B**); 0.10 mm (**C, D**).

**Figure 3.**
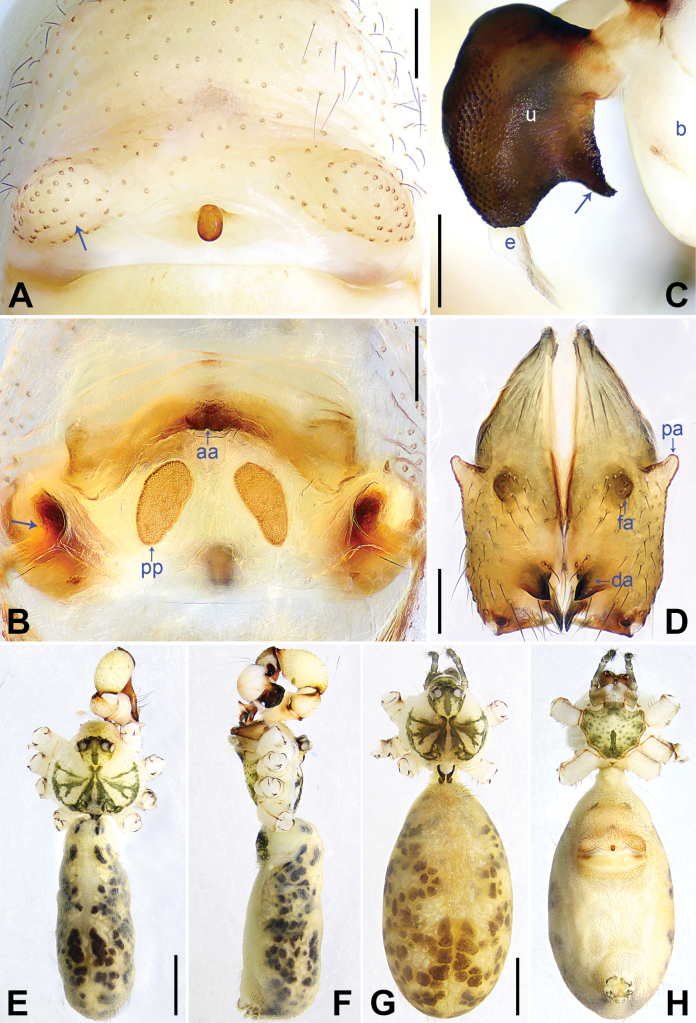
*Pholcusfengmeii* sp. nov., holotype male (**C–F**) and paratype female (**A, B, G, H**) **A** epigyne, ventral view, arrow indicates lateral protrusion **B** vulva, dorsal view, arrow indicates lateral sclerite **C** bulbal apophyses, prolateral view, arrow indicates latero-median protrusion **D** chelicerae, frontal view **E–H** habitus (**E, G** dorsal view **F** lateral view **H** ventral view). Abbreviations: aa = anterior arch, b = bulb, da = distal apophysis, e = embolus, fa = frontal apophysis, pa = proximo-lateral apophysis, pp = pore plate, u = uncus. Scale bars: 0.20 mm (**A–D**); 1.00 mm (**E–H**).

**Figure 4.**
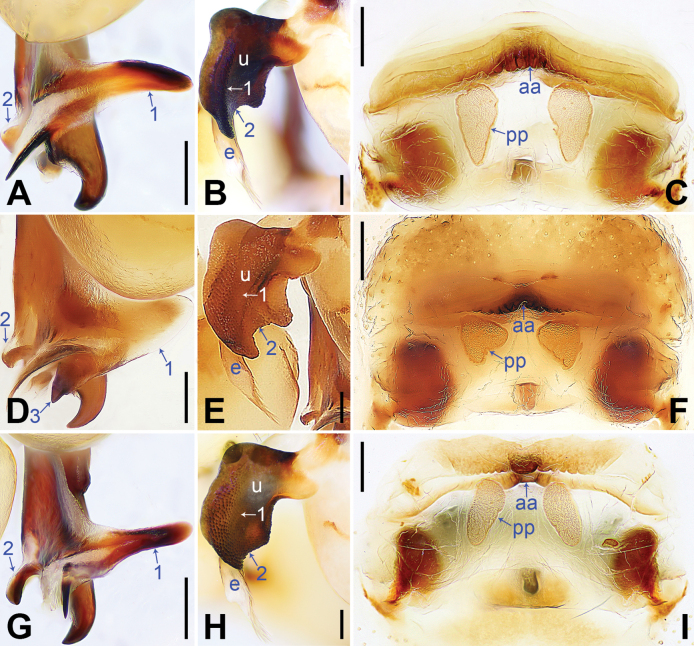
*Pholcusjiguanshan* (**A–C**), *P.phoenixus* (**D–F**), *P.yaoshan* (**G–I**) **A, D, G** distal parts of procursus, prolateral views, arrows 1 indicate prolatero-subdistal sclerite, arrows 2 indicate ventro-subdistal apophysis, arrow 3 indicates angular proximal apophysis **B, E, H** bulbal apophyses, prolateral views, arrows 1 indicate median part, arrows 2 indicate strongly/slightly curved distal part **C, F, I** vulvae, dorsal views. Abbreviations: aa = anterior arch, e = embolus, pp = pore plate, u = uncus. Scale bars: 0.10 mm (**A, B, D, E, G, H**); 0.20 mm (**C, F, I**).

The new species resembles *P.phoenixus* (Fig. 4D–F; Yao and Li 2012: figs 144A–D, 145A–C) by having similar epigyne (Fig. 3A) and male chelicerae (Fig. 3D), but it can be easily distinguished by procursus lacking ventro-subdistal apophysis (Fig. 2C; vs present, arrow 2 in Fig. 4D), by prolatero-subdistal sclerite of procursus lacking angular proximal apophysis (Fig. 2C; vs present, arrow 3 in Fig. 4D), by uncus nearly half-round, with latero-median protrusion (arrow in Fig. 3C; vs with latero-median and distal protrusions, Fig. 4E), by anterior arch medially strongly curved (bow-shaped, Fig. 3B; vs straight, Fig. 4F), and by pore plates nearly half-round (Fig. 3B; vs nearly triangular, Fig. 4F).

#### Description.

**Male** (***holotype***): Habitus as in Fig. 3E, F. Total length 4.85 (5.08 with clypeus), carapace 1.41 long, 1.62 wide, opisthosoma 3.44 long, 1.34 wide. Legs: I: 36.90 (9.55, 0.74, 9.04, 15.19, 2.38), II: 25.06 (6.99, 0.64, 6.22, 9.68, 1.53), III: 17.98 (5.19, 0.60, 4.17, 6.79, 1.23), IV: 23.47 (6.79, 0.68, 5.77, 8.85, 1.38); tibia I L/d: 65. Eye interdistances and sizes: PME–PME 0.26, PME 0.15, PME–ALE 0.04, AME–AME 0.06, AME 0.10. Sternum width/length: 1.10/0.97. Carapace yellowish, with brown radiating marks and marginal brown bands; ocular area yellowish, with median and lateral brown bands; clypeus yellowish, with brown median marks; sternum yellowish, with brown marks and posterior median stripe. Legs yellowish, but dark brown on patellae and whitish on distal parts of femora and tibiae, with darker rings on subdistal parts of femora and proximal and subdistal parts of tibiae. Opisthosoma yellowish, with dorsal and lateral spots. Chelicerae (Fig. 3D) with pair of proximo-lateral apophyses, pair of distal apophyses with 2 teeth each, and pair of frontal apophyses. Legs with short erected setae on tibiae, metatarsi, and tarsi; retrolateral trichobothrium on tibia I at 3% proximally; tarsus I with 33 distinct pseudosegments.

Palp as in Fig. 2A, B; trochanter with long (4 times longer than wide), retrolaterally strongly bulged ventral apophysis (arrow 1 in Fig. 2B); femur with small retrolatero-proximal protrusion (arrow 2 in Fig. 2B) and indistinct ventral protrusion; tibia with prolatero-ventral protrusion (arrow in Fig. 2A); procursus simple proximally and complex distally, with proximally wide prolatero-subdistal sclerite (arrow 1 in Fig. 2C) equipped with curved proximal apophysis (arrow 2 in Fig. 2C), curved distal apophysis (arrow 3 in Fig. 2C), and 3 dorsal spines (arrow in Fig. 2D); uncus nearly half-round, 1.3 times longer than wide, with scales and latero-median protrusion (arrow in Fig. 3C); bulb without appendix; embolus weakly sclerotized, with some indistinct transparent distal projections (Fig. 3C).

**Female** (***paratype***, SYNU-Ar00360): Similar to male, habitus as in Fig. 3G, H. Total length 5.83 (5.99 with clypeus), carapace 1.45 long, 1.68 wide, opisthosoma 4.38 long, 2.33 wide; tibia I: 8.85; tibia I L/d: 52. Eye interdistances and sizes: PME–PME 0.22, PME 0.15, PME–ALE 0.05, AME–AME 0.06, AME 0.09. Sternum width/length: 1.11/0.95. Clypeus brown.

Epigyne (Fig. 3A) 1.2 times wider than long, with antero-median brownish marks, short oval knob (1.6 times longer than wide), and pair of lateral protrusions anterior to epigynal plate (arrow in Fig. 3A). Vulva (Fig. 3B) with strongly curved, sclerotized anterior arch, pair of nearly half-round pore plates (2 times longer than wide), and pair of curved lateral sclerites (arrow in Fig. 3B).

#### Variation.

Tibia I in two paratype males (SYNU-Ar00358–59): 10.51, 10.90. Tibia I in the other two paratype females (SYNU-Ar00361–62): 7.76, 8.01.

#### Natural history.

Specimens were found on the underside of overhang on rocky outcrop and wooden railings in rural areas.

#### Distribution.

China (Liaoning, type locality; Fig. 1).

## Supplementary Material

XML Treatment for Pholcus

XML Treatment for Pholcus fengmeii

## ﻿Additional information

### Author ORCIDs

Ludan Zhang https://orcid.org/0000-0001-5657-751X

Bing Wang https://orcid.org/0009-0009-7047-4395

Qiaoqiao He https://orcid.org/0000-0002-9381-7444

Zhiyuan Yao https://orcid.org/0000-0002-1631-0949

### Data availability

All of the data that support the findings of this study are available in the main text.
